# Memory in multiple sclerosis is linked to glutamate concentration in grey matter regions

**DOI:** 10.1136/jnnp-2013-306662

**Published:** 2014-01-15

**Authors:** Nils Muhlert, Matteo Atzori, Enrico De Vita, David L Thomas, Rebecca S Samson, Claudia A M Wheeler-Kingshott, Jeroen J G Geurts, David H Miller, Alan J Thompson, Olga Ciccarelli

**Affiliations:** 1 Department of Neuroinflammation, NMR Research Unit, UCL Institute of Neurology, London, UK; 2 Cognitive Neuroscience, Department of Psychology, Cardiff University, Cardiff, UK; 3 Department of Brain Repair and Rehabilitation, NMR Research Unit, UCL Institute of Neurology, London, UK; 4 Department of Neurology, University of Padova, Padova, Italy; 5 Lysholm Department of Neuroradiology, National Hospital for Neurology and Neurosurgery, London, UK; 6 Neuroradiological Academic Unit, Department of Brain Repair and Rehabilitation, UCL Institute of Neurology, London, UK; 7 Department of Anatomy and Neuroscience, Section of Clinical Neuroscience, VU University Medical Center, VUmc MS Center Amsterdam, Amsterdam, The Netherlands; 8 National Institute for Health and Research (NIHR) University College London Hospital (UCLH) Biomedical Research Centre, London, UK

## Abstract

**Objective:**

Glutamate is the principal excitatory neurotransmitter and is involved in normal brain function. Cognitive impairment is common in multiple sclerosis (MS), and understanding its mechanisms is crucial for developing effective treatments. We used structural and metabolic brain imaging to test two hypotheses: (i) glutamate levels in grey matter regions are abnormal in MS, and (ii) patients show a relationship between glutamate concentration and memory performance.

**Methods:**

Eighteen patients with relapsing-remitting MS and 17 healthy controls were cognitively assessed and underwent ^1^H-magnetic resonance spectroscopy at 3 T to assess glutamate levels in the hippocampus, thalamus, cingulate and parietal cortices. Regression models investigated the association between glutamate concentration and memory performance independently of magnetisation transfer ratio values and grey matter lesions withint he same regions, and whole-brain grey matter volume.

**Results:**

Patients had worse visual and verbal memory than controls. A positive relationship between glutamate levels in the hippocampal, thalamic and cingulate regions and visuospatial memory was detected in patients, but not in healthy controls.

**Conclusions:**

The relationship between memory and glutamate concentration, which is unique to MS patients, suggests the reliance of memory on glutamatergic systems in MS.

## Introduction

Cognitive impairment is common in multiple sclerosis (MS), occurs at all stages of the disease and greatly impacts on patients’ lives.^1^ Memory is frequently affected and has been linked to both widespread grey matter (GM) cortical thinning^2^ and atrophy in specific GM regions, such as the hippocampus.^3^ MRI-derived GM atrophy may reflect neuronal loss, demyelination or reduced synapse or glial densities.^4^ Neuronal loss and demyelination can be prominent features in the hippocampi of MS patients.^5^ Demyelinated MS hippocampi also show reductions in synaptic density and in the neuronal proteins essential for axonal transport, synaptic plasticity, glutamate neurotransmission, glutamate homeostasis and memory, relative to normally myelinated hippocampi.^6^ Similarly, reductions in excitatory amino acid transporters, which carry glutamate from the synaptic cleft to astrocytes, have been observed.^7^ These postmortem findings implicate abnormal glutamate neurotransmission in cognitive impairment in MS.

Current imaging techniques do not allow direct measurement of glutamate neurotransmission because several of its components are not detectable in vivo, including the functioning of glutamate receptors and transporters, glutamate–glutamine cycling and synaptic activity. However, estimates of the total concentration of glutamate (and its precursor glutamine) can be obtained with in vivo MR spectroscopy (MRS). Although the availability of the neurotransmitter pool cannot be measured in vivo, the concentration of glutamate inside the nerve terminal cells is several thousand times higher than in the extracellular fluid. MRS studies in patients with schizophrenia have demonstrated reduced glutamate levels and increased glutamine concentration in the medial prefrontal region,^8^ suggesting aberrant glutamatergic processes in schizophrenia. In people with MS, MRS studies have shown increased glutamate levels in active lesions and normal-appearing white matter (WM), suggesting possible mechanisms of glutamate-mediated excitotoxicity, but normal levels in chronic lesions, compared with controls.^9^ The effect of MS on levels of glutamate in the GM remains unknown.

In this study, we address two hypotheses: (i) glutamate levels in hippocampal, thalamic, cingulate and parietal GM regions are abnormal in patients with relapsing-remitting (RR) MS; (ii) there is an association between glutamate levels in GM regions and memory function. In particular, we predicted that visuospatial memory would be related to glutamate in these regions, given its links with the right hippocampus,^10^ and previous links with thalamic integrity in people with MS.^11^ ^12^ We also investigated whether this association was independent of imaging measures derived from the same areas, reflecting structural damage (such as demyelination and reduced axonal density).

## Materials and methods

### Subjects

Eighteen patients with a diagnosis of RRMS^13^ and without a history of relapse or treatment with corticosteroids within the preceding 4 weeks were recruited. Seventeen age-matched and gender-matched healthy volunteers were also studied. Matching was carried out at group level and not on a case-by-case basis. Written informed consent was obtained for participation in the study, which was approved by our local ethics committee.

### Cognitive tests

Visuospatial learning and memory was assessed using the Paired Associates Learning (PAL) test from the CANTAB (Cambridge Cognition, Cambridge, UK). In this test, participants have to remember the location of patterns presented in different areas of the screen. The number of patterns increases over a series of trials from two to eight patterns. Two age-scaled z-scores are produced based on standardised normative data: the number of trials completed at the first attempt and the total number of trials needed to complete the test. To assess cognition generally, we also assessed verbal learning and memory using list-learning from the Adult Memory and Information Processing Battery (AMIPB) and working memory with the digit-span from the Wechsler Adult Intelligence Scale-III.^14^ Speed of information processing was assessed using the oral version of the Symbol-Digit Modalities Test (SDMT), for which z-scores were obtained with reference to published norms.^15^ Executive function was measured using the Stroop colour-word interference test.^16^

Premorbid IQ was measured using the National Adult Reading Test, and current IQ was measured with the two-subtest version of the Wechsler Abbreviated Scale of Intelligence (vocabulary and matrix reasoning). Levels of anxiety and depression were measured with the Hospital Anxiety and Depression Scale.^17^

Failure of a test (ie, PAL trials at first attempt, PAL total trials, AMIPB list learning, AMIPB delayed recall, SDMT, Stroop) was defined as a score at two or more SDs below the mean of the controls. Patients with significant cognitive impairment were defined as those showing failure on at least two tests.

### MRI protocol and analysis

Imaging was performed on a 3 T Siemens Tim Trio scanner with a 32-channel head coil.

#### Magnetisation Transfer (MT) imaging

Sagittal volumes were acquired at 1×1×1 mm^3^ (FOV 240×256×176 mm^3^; phase encoding direction superior/inferior; GRAPPA acceleration factor 2). A slab selective 3D multiecho FLASH sequence (repetition time (TR)=23.7 ms, echo time (TE)=2.2 ms, flip angle (α)=6°) was performed twice, with either predominantly PD-weighting (eight equally spaced echoes with a TE difference of 2.5 ms) or MT-weighting (with an additional 4 ms off-resonance Gaussian radiofrequency (RF) pulse (nominal α=220°, offset frequency 2 kHz) before each excitation pulse, six equally spaced echoes^18^). The multiecho data were averaged (for the PD-weighted images, the first six echoes only were averaged) and the resulting MT-w (MTon) and PD-w (MToff) images were coregistered to the T1-weighted volume prior to calculation of the magnetisation transfer ratio (MTR) maps (MTR=MToff – MTon/MToff). MTR maps were masked using the spectroscopic volumes-of-interest (VOIs), and the mean MTR was calculated within each VOI.

#### Single-voxel spectroscopy

**Figure 1:**
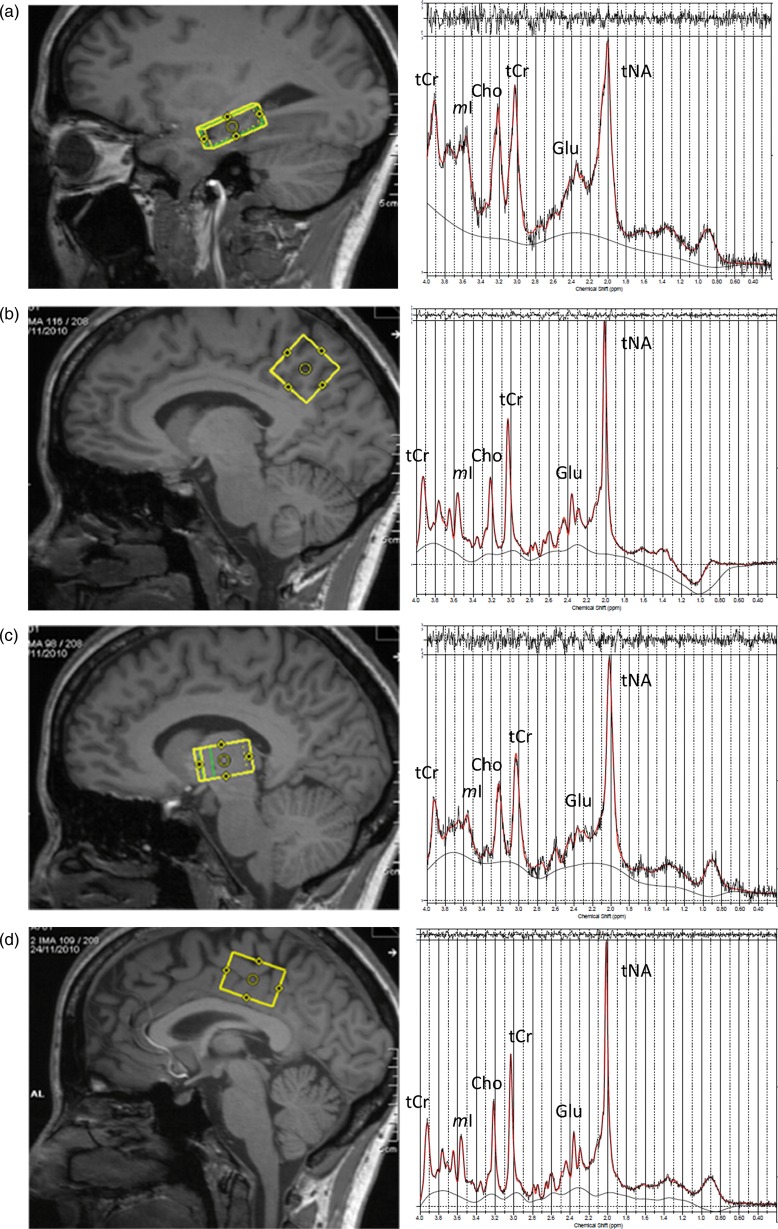
Placement of MR spectroscopy (MRS) voxels (left) with their example MRS spectra (right) in the right hippocampus (A), parietal cortices (B), right thalamus (C) and cingulate (D).

A single-voxel PRESS with chemical selective saturation water suppression was used (TR=6000 ms, TE=30 ms). The long TR was chosen to minimise the potential effect of pathological T1 changes on the acquired spectra. The dimensions and averages for the VOI were as follows: (1) right hippocampus: dimensions 26×16×16 mm^3^, VOI volume=7.3 mL, 144 averages; (2) right thalamus: dimensions 26×16×16 mm^3^, VOI volume=6.7 mL, 144 averages; (3) posterior cingulate cortex: dimensions 30×25×16 mm^3^, VOI volume=12 mL, 48 averages; and (4) medial posterior parietal cortex: dimensions 25×25×25 mm^3^, VOI volume=15.6 mL, 48 averages. Example placements of each VOI can be seen in figure 1. A non-water-suppressed spectrum was also acquired with the same parameters (two averages) to provide an internal water reference to scale the measured metabolite signals.

The spectra were analysed using LCModel 6.1.^19^ LCModel was used to estimate the concentrations of glutamate ([Glu]), glutamate plus glutamine ([Glx]), total *N*-acetyl-aspartate and *N*-acetyl-aspartate glutamate ([tNA]), choline-containing compounds ([Cho]), creatine-plus-phosphocreatine ([tCr]) and *myo*-Inositol ([*m*Ins]), which were scaled using the water signal from a non-water-suppressed spectrum. All spectra were checked for quality. The right hippocampal spectrum was not acquired in one patient, and the cingulate spectrum was not acquired in one control due to time constraints. Four patients and two controls had poor hippocampal spectra and two patients had poor thalamic spectra, with signal to noise ratios (SNR) less than 10. These were excluded from the analyses. Reanalysing data with all participants included did not affect the pattern of results. Example spectra for each VOI in a control participant can be seen in figure 1.

The GM, WM and cerebrospinal fluid (CSF) masks obtained by segmentation of the T1 scans were used to calculate the fractional tissue content of each VOI. With these, it was possible to estimate the water concentration within each VOI and estimate the amplitude of the water signal in each VOI. We also used literature-based values of T1 and T2 relaxation times of GM, WM and CSF for each VOI, and the sequence parameters (TE and TR), to correct the LCModel output for VOI water content (see online supplementary information for further details). Estimated metabolite concentrations thus corrected are reported in ‘institutional units’ (iu).

#### Conventional imaging

Participants underwent axial T2-weighted imaging (turbo spin–echo (TSE) with spatial resolution of 0.9×0.9×3 mm^3^, TR=5760 ms, TE=101 ms), which was used to mark T2-hyperintense lesions, and 3D T1-weighted imaging (magnetisation-prepared rapid gradient echo with spatial resolution of 0.9×0.9×0.9 mm^3^, inversion time (TI)=900 ms, TR=1900 ms, TE=2.81 ms). Hypointense lesions on the T1-weighted volume were marked and filled using the average WM intensity^20^ before segmentation using the ‘new_segment’ function in SPM8 (http://www.fil.ion.ucl.ac.uk/spm/software/spm8), which reduces the impact of lesion-associated segmentation bias on GM and WM segmentations.^20^ Intracranial volumes were defined in each subject by first using a maximum likelihood algorithm to binarise the GM, WM and CSF tissue volumes and then summing each of these volumes.

#### Double inversion recovery lesions

All subjects also underwent 3D double inversion recovery (DIR) imaging (using an optimised long echo train TSE readout with spatial resolution of 1.3×1.2×1.2 mm^3^, TR=10 000 ms, TE=274 ms, TI=3000/510 ms). DIR lesions were marked for each patient, following recent consensus recommendations.^21^ In order to count the number of DIR lesions within each spectroscopic voxel, T1-weighted scans were rigidly registered to DIR scans; the resulting transformation parameters were then applied to the MRS voxels to transform them into DIR space.

### Statistical analyses

#### Differences in cognitive performance and metabolite concentrations between groups

Differences between all patients (and cognitively impaired (CI) and cognitively preserved (CP) patients, see online supplementary information) and controls in demographics, neuropsychological test performance, MTR values in the spectroscopic regions and in the GM or WM volume fractions in the VOIs were compared using independent-sample t tests. Group differences in [Glu] (and other metabolites) were initially tested using a repeated measures multivariate analysis of variance (MANOVA) with factors of group (patient vs control) and spectroscopic VOI (hippocampus, thalamus, cingulate and parietal cortices), therefore applying family-wise correction for multiple comparisons. The repeated measures MANOVA was used to examine group differences in metabolites and interactions between groups and regions. When the MANOVA showed significant group differences across all four VOIs, group differences were then tested in each VOI using independent samples t tests. A significance threshold of p<0.05 was used throughout.

#### Association between cognitive performance and regional glutamate levels in patients and controls

Associations between [Glu] and cognitive performance in all patients were investigated using three linear regression models, each with different independent variables. In the first, the [Glu] of each region was entered into the model as predictor, and visuospatial memory as the outcome variable. The same analyses were repeated in controls.

When a significant association (or a trend towards significance) between [Glu] and cognitive test performance was found, a second linear regression model was run, entering the [Glu] of a specific VOI, the DIR GM lesions within the spectroscopic VOI and the MTR value of the VOI, as predictors, and the cognitive test performance as the dependent variable. This aimed at identifying whether [Glu] was associated with memory performance independently from MTR and GM lesions. The partial correlation coefficients (PCCs), which give a measure of the relative strengths of the independent associations between predictors and dependent variable, were reported when more than one predictor was found to be significant. Finally, the regression analyses in which [Glu] significantly predicted memory performance were repeated, including whole-brain GM volume fraction as predictor, to assess whether GM volume mediated the effect. All statistical analyses were carried out using the statistical package for social scientists (SPSS) V.20.0. An α level of p<0.05 was used throughout, and significance values less than 0.10 were considered trends.

To complete this study, the link between [Glu] and [tNA] and between [tNA] and memory was assessed in patients; methods and results for this section are described in the online supplementary information.

## Results

### Demographics, clinical and cognitive results

The demographic and clinical characteristics of all subject groups are shown in table 1. Taken together, all patients showed worse visuospatial memory than controls on the PAL test, with fewer trials completed at the first attempt (t=2.4, p<0.05) and fewer trials completed overall (t=3.0, p<0.01) (table 1). Patients also had worse verbal memory than controls, with worse learning of the list (t=3.3, p<0.01), fewer words recalled after distraction (t=3.2, p<0.01) and fewer words recalled after a 30 min delay (t=2.2, p<0.05). Patients had slower processing speed than controls on the SDMT (t=4.7, p<0.001) and were worse on the Stroop task, suggesting impaired executive function (t=−4.0, p<0.01) (table 1). In contrast, patients and controls did not differ on working memory (t=1.7, p=0.10) (table 1). Additionally, patients did not differ from controls in their age, their premorbid IQ or their current IQ; the two groups showed no significant differences in levels of anxiety or depression (table 1).

**Table 1:** Mean (SD) demographics and cognitive performance for patients and controls

Demographic and clinical characteristics	Controls	Patients
N	17	18
Age (SD, median, range)	39.7 (11.1, 40.0, 29–60)	43.5 (8.5, 44.0, 31–57)
Gender (M:F)	11:6	12:6
EDSS (median (range))	–	3.25 (1.0–6.5)
Premorbid IQ	108.6 (11.7)	105.2 (13.4)
Current IQ	112.6 (16.8)	110.4 (12.9)
Anxiety	5.3 (2.8)	5.4 (3.9)
Depression	2.7 (3.5)	3.9 (3.2)
Paired associate learning		
Trials at first attempt, z-score	0.2 (1.3)	−0.7 (1.1)*
Total trials, z-score	0.2 (1.0)	−0.8 (0.9)**
List learning		
Learning (total words)	57.7 (9.0)	47.2 (9.3)**
Recall after distraction (words recalled)	12.2 (2.2)	9.2 (3.1)**
30 min delayed recall (words recalled)	11.8 (2.4)	9.7 (3.2)*
Digit span (total score)	18.4 (3.1)	16.6 (3.3)
Stroop (seconds to complete)	101.7 (12.3)	150.5 (49.2)**
SDMT, z-score	0.9 (0.8)	−0.9 (1.2)***

Asterisks indicate significantly different results between patients and controls using independent samples t tests (*p<0.05, **p<0.01, ***p<0.001).

EDSS, expanded disability status scale; SDMT, symbol-digit modalities test.

### Glutamate levels in GM regions are lower in MS patients than controls

**Figure 2:**
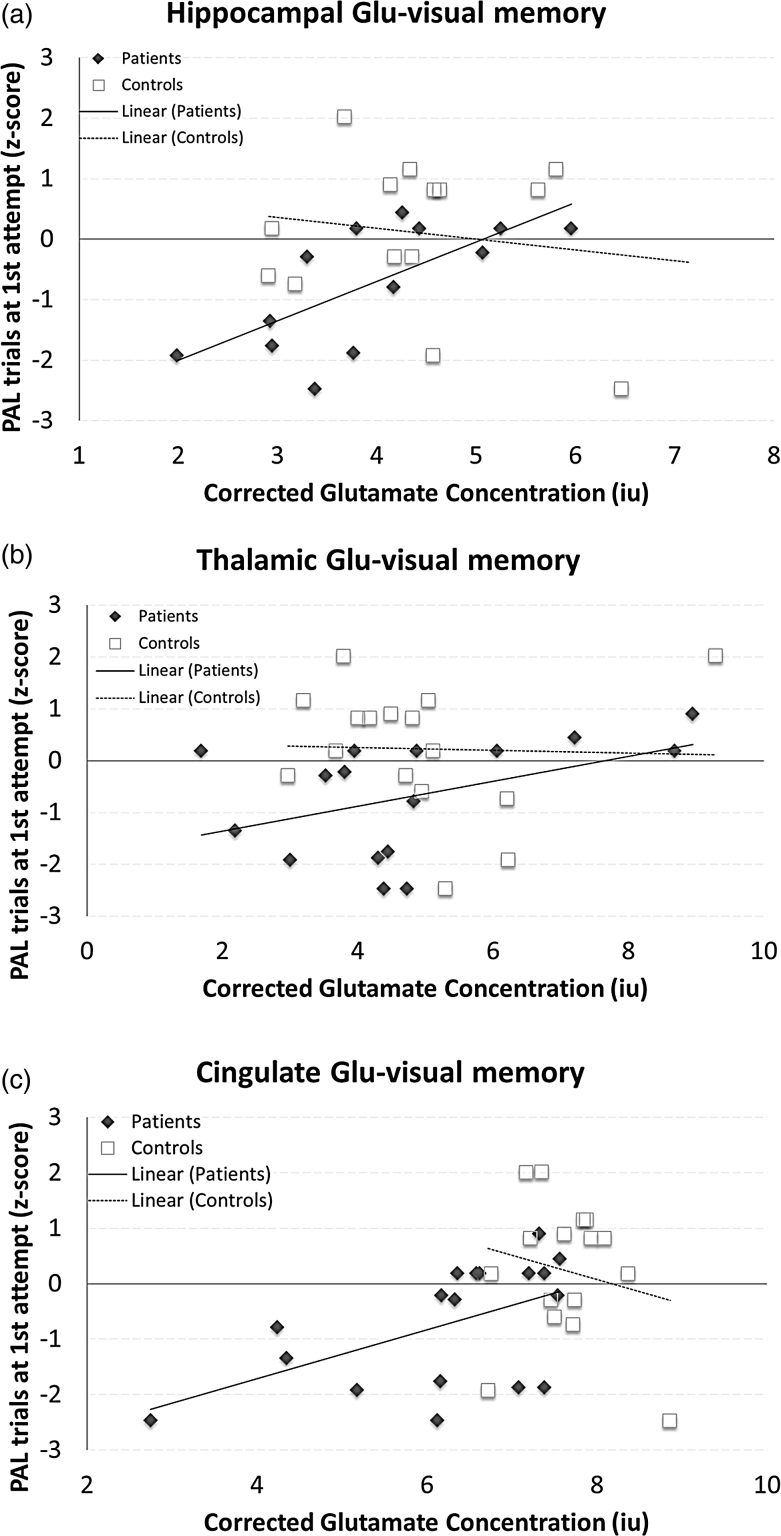
Three scatter plots showing the associations and line of best fit between memory function and [Glu] in the hippocampus (A), thalamus (B) and cingulate (C) for patients (black diamonds and solid line) and controls (open squares and dotted line).

The MANOVA analysis showed that all patients together had significantly lower [Glu] (and [Glx]) in GM regions than controls ([Glu]: Pillai's trace=0.5, F=5.684, p<0.01; [Glx]: Pillai's trace=0.4, F=3.53, p<0.05). There was no significant interaction between group and region for [Glu] (p=0.18) or [Glx] (p=0.56). Subsequent univariate analyses in each individual VOI revealed that the patient group had significantly lower [Glu] (and [Glx]) in the cingulate and parietal cortices (all p<0.01), and a trend towards lower [Glu] in the right hippocampus (p=0.10) when compared with controls (table 2, figure 2). To complete the study, the differences between all patients and controls in [tNA] and [tCr] were examined and results are reported in the online supplementary information.

**Table 2:** Mean (SD) glutamate ([Glu]) and glutamate and glutamine ([Glx]) concentration estimates in institutional units for patients and controls

	Controls	Patients
Hippocampus		
Glu	4.57 (1.2)	3.94 (1.1)
Glu %SD	8.85 (2.5)	9.85 (3.8)
Glx	5.19 (1.5)	4.48 (1.5)
Glx %SD	10.36 (3.4)	11.23 (4.0)
Thalamus		
Glu	4.99 (1.5)	4.79 (2.1)
Glu %SD	12.35 (3.60)	11.47 (3.5)
Glx	5.84 (2.4)	6.05 (3.9)
Glx %SD	12.56 (3.8)	13.35 (4.0)
Cingulate cortex		
Glu	7.60 (0.6)	6.24 (1.3)*
Glu %SD	6.12 (0.8)	6.72 (1.4)
Glx	8.31 (0.8)	6.94 (1.5)*
Glx %SD	6.71 (1.3)	7.22 (1.3)
Parietal cortex		
Glu	7.44 (0.7)	6.30 (1.1)*
Glu %SD	5.35 (0.9)	5.89 (1.5)
Glx	8.49 (0.8)	7.21 (1.2)*
Glx %SD	5.58 (0.7)	6.39 (1.6)

*p<0.01 for patients versus controls comparison.

On the other measures of structural damage calculated within the spectroscopic voxels, all patients together showed significantly lower MTR than controls in the thalamic, cingulate and parietal regions, but no group differences in GM or WM volume fractions were observed (table 3).

**Table 3:** T2 lesion load and total and regional DIR GM lesions of patients, and MTR values within the spectroscopic voxels of patients and controls

	Controls	Patients
T2 lesion load (mean (SD))	–	7.05 mL (7.60)
DIR lesions (median (range))		
Total grey matter lesions	–	22 lesions (range 6–275)
Hippocampal lesions in the right hippocampal spectroscopic VOI	–	0.5 lesions (range 0–3)
Thalamic lesions in the right thalamic VOI	–	0 lesions (range 0–2)
Grey matter lesions in the cingulate cortex VOI	–	0 lesions (range 0–12)
Grey matter lesions in the post. parietal VOI	–	1 lesion (range 0–6)
Total GM lesions in each structure on DIR (median[range])		
Right hippocampal lesions		0.5 lesions (range 0–3)
Right thalamic lesions		0 lesions (range 0–2)
GM fraction (mean (SD))	0.45 (0.04)	0.42 (0.02)*
MTR (mean (SD))		
MTR in the right hippocampal VOI	33.1 (2.0)	31.1 (4.4)
MTR in the right thalamic VOI	30.8 (1.5)	29.1 (2.8)*
MTR in the cingulate cortex VOI	30.9 (1.1)	28.6 (2.7)*
MTR in the posterior parietal VOI	37.9 (0.9)	35.3 (2.5)*

Note that the number of DIR lesions in the patients’ hippocampus and thalamus was similar to those seen in the spectroscopic hippocampal and thalamic voxels.

*p<0.05.

DIR, double inversion recovery; GM, grey matter; MTR, magnetisation transfer ratio; VOI, spectroscopic volume of interest; WM, white matter.

### Associations between imaging measures and cognitive impairment

Memory was significantly associated with [Glu] in the hippocampus, thalamus and cingulate VOIs in all patients, as reported below (figure 2). Statistically significant results in patients did not change after correcting the models for whole-brain GM volume fraction. Healthy controls did not show significant associations between memory and [Glu] in any of the GM regions (figure 2).

#### Hippocampus

PAL trials completed at first attempt were significantly predicted by hippocampal [Glu] (coeff.=0.69, 95% CI 0.20 to 1.09, p=0.008, PCC=0.69). Entering hippocampal MTR and hippocampal DIR lesions as predictors revealed that only hippocampal [Glu] showed a trend for predicting PAL trials at first attempt (coeff.=0.60, 95% CI −0.01 to 1.13, p=0.054, PCC=0.59).

There was a trend towards a significant association between PAL total trials and hippocampal [Glu] (coeff.=0.55, 95% CI −0.01 to 0.64, p=0.051, PCC=0.55). This trend became significant after correcting for hippocampal MTR and hippocampal DIR lesions (coeff.=0.71, 95% CI 0.01 to 0.82, p=0.049, PCC=0.60).

#### Thalamus

There was a trend towards a significant association between PAL trials at first attempt and thalamic [Glu] (coeff.=0.24, 95% CI −0.04 to 0.52, p=0.088). Entering thalamic MTR and thalamic DIR lesions to the model revealed that thalamic [Glu] and thalamic DIR lesions significantly predicted PAL trials at first attempt ([Glu]: coeff.=0.53, 95% CI 0.09 to 0.49, p=0.009, PCC=0.70; DIR lesions: coeff.=−0.68, 95% CI −1.09 to −0.26, p=0.004, PCC=−0.71).

PAL total trials were not associated with thalamic [Glu].

#### Cingulate cortex

PAL trials at first attempt were significantly predicted by cingulate [Glu] (coeff.=0.54, 95% CI 0.07 to 0.80, p=0.022). Adding cingulate MTR and cingulate DIR lesions revealed that cingulate [Glu] and cingulate DIR lesions significantly predicted PAL at first trial ([Glu]: coeff.=0.50, 95% CI 0.02 to 0.79, p=0.038, PCC=0.52; DIR lesions: coeff.=−0.46, 95% CI −0.35 to −0.01, p=0.037, PCC=−0.52).

PAL total trials were not associated with cingulate [Glu].

#### Parietal cortex

Parietal [Glu] did not predict memory.

### Correcting for GM and WM fraction

Repeating the regression models after including GM fraction and WM fraction did not change the pattern of results.

## Discussion

We provide support for (i) MS-related modifications of glutamate levels in specific GM regions and (ii) positive correlations between glutamate levels and visuospatial memory performance in MS. The same associations are not detected in healthy controls. We discuss these two findings in turn.

When all patients were grouped together, glutamate was significantly lower in patients than healthy controls in the parietal and cingulate regions, and there was a trend towards lower glutamate in the right hippocampus in patients. Glutamate–glutamine levels were similarly reduced in patients compared with controls, indicating an overall reduction in glutamatergic activity, since glutamine is the precursor of glutamate. MRS quantification of glutamate concentrations does not allow us to draw firm conclusions about the mechanisms underlying the observed reductions, as many of the components of glutamate-mediated neurotransmission may be implicated and these are not detectable in vivo. Glutamate is much more abundant inside cells than in the extracellular space, and its highest concentrations are found in the synaptic vesicles. Therefore, a possible interpretation is that reduced synapses and neuronal–axonal degeneration, which have been described in histological analyses of MS brains,^22^ may be relevant for determining lower glutamate levels. Along the same lines, reduced glutamate, possibly associated with shrinkage/neuronal loss, has been described during the ageing process^23^ and in schizophrenia.^8^ Finally, the observed (and positive) correlations between glutamate and tNA in the hippocampal, cingulate and parietal regions suggest that reduced neuronal integrity and/or metabolism, reflected by reduced tNA,^24^ may underlie the same pathological processes reflected by reduced glutamate levels.

Considering the thalamic and hippocampal regions, no group differences in glutamate and glutamate–glutamine were found in the thalamic voxel, and a trend towards statistical significance was detected in the hippocampus. This may reflect the unique tissue microstructure of the thalamus when compared with cortical regions (the thalamus is a striated GM and WM structure) and be related to the greater technical challenge of segmenting the thalamic region with SPM8,^25^ and of acquiring spectra in the thalamus and, to a lesser extent, the hippocampus.^26^ Yet, whereas previous studies carried out at lower field strengths (1.5 T) have generally considered glutamate and glutamine together, we were able to identify the glutamate resonance with an acceptable level of confidence. We also improved the accuracy of our metabolite concentrations by adjusting them for water content of GM, WM and CSF and for regional T1 and T2 values.

When looking at the associations between glutamate concentrations and cognitive tests, we found that, in patients, visuospatial memory was significantly associated with hippocampal glutamate independently of MTR and DIR GM lesions within the same region. Additionally, in patients, visuospatial memory was more strongly predicted by cingulate glutamate than by DIR lesions within the cingulate. Worse visuospatial memory in patients was also predicted by lower glutamate in the thalamic region, although thalamic DIR lesions were equally (and independently) predictive. All these associations remained significant after adjusting for whole-brain GM volume fraction. Since MTR is sensitive to cortical demyelination,^27^ while DIR lesions reflect focal areas of demyelination,^28^ these findings suggest that the association between memory and glutamate levels may not depend on cortical demyelination or overall GM atrophy. Overall, these findings suggest that (i) glutamate levels in these GM regions reflect processes that are linked with memory function in MS; these processes are unlikely to occur in the general population given that these correlations were not found in healthy controls. (ii) DIR GM lesions in the thalamic region may also affect memory function, which confirms previously published reports on the association between thalamic lesions and atrophy and memory function in MS.^11^

In schizophrenia, reduced cortical glutamate in frontal regions is known to be associated with impaired cognition,^29^ and cognition is impaired in healthy humans given NMDA antagonists.^30^ Both CI and CP patients showed similar reductions in glutamate levels in the parietal and cingulate GM regions when compared with controls (overall differences were between 15% and 22%, see online supplementary information), suggesting that glutamate levels reflect MS-related pathological processes, rather than a dysfunction that specifically mediates cognitive impairment. Future work will aim to examine whether people with MS also show changes in glutamate levels in other GM regions that are known to contribute to cognitive function, such as the frontal regions and subcortical GM nuclei, and whether such changes help to explain cognitive impairment.

In contrast to glutamate, tNA did not significantly predict memory. This study did not aim to assess the comparative predictive values of glutamate and tNA, and given their strong correlation, it would have been underpowered to do so.

Our study has some unavoidable limitations. First, because of the cuboidal shape of the spectroscopic VOIs and the linear relation between VOI volume and spectroscopic SNR, in order to achieve reliable estimates of metabolite concentrations in reasonable acquisition times, it was necessary to have VOIs that encompassed the GM of interest rather than being completely contained within it. Therefore, inevitably these VOIs included some of the WM surrounding the regions of interest. However, patients and controls showed no differences in the GM and WM volume fractions, the regressions were generally unaffected after accounting for GM and WM fractions and fractions were used to adjust the glutamate concentration for water content of both tissue types. Additionally, the relationship between glutamate levels and cognitive performance did not change after correcting for whole-brain GM volume fraction. Second, 16 regressions were performed without formal correction for multiple comparisons. This can be justified by the fact that this is a hypothesis-driven rather than an exploratory study, making the need for multiple comparison corrections less relevant.^31^ In this case, controlling for type II errors is as important as controlling for type I errors. Finally, while the study was powered to assess differences in glutamate between patients and controls, it was not powered to assess differences between CI and CP patients. These data however answer an interesting posthoc question that can be explored in future work.

## Conclusion

Using a MRS protocol optimised for glutamate detection, but widely available at 3 T, this study provides the first evidence that MS affects glutamate levels in GM and that worse visuospatial memory is linked to lower glutamate in the hippocampal, thalamic and cingulate regions in RRMS patients. As in other neuropsychiatric diseases,^29^ the discovery of a link between glutamate abnormalities in the GM of MS patients and reduced memory performance may lead to new treatment approaches that specifically modulate this neurotransmitter.

## Supplementary Material

Web supplement
