# Supplementary material for: Memory in multiple sclerosis is linked to glutamate concentration in grey matter regions
Source: J Neurol Neurosurg Psychiatry. 2014 Jan 15;85(8):833–9. doi: 10.1136/jnnp-2013-306662 (PMC4112488; doi:10.1136/jnnp-2013-306662)
Supplement: Web supplement [file jnnp-2013-306662-s1.pdf]

Supplementary Information:

(1) LCModel output correction

Correction of LCModel outputs

The GM, white matter and CSF masks obtained by segmentation of the T1 scans were used to calculate the fractional tissue content of each spectroscopic VOI. With these, the LCModel output was corrected for the water signal adjusted for brain water content. The output was also corrected for T1 and T2 relaxation times for each region. The NMR visible water concentration in a spectroscopic voxel, based on Ernst and colleagues,[S1] are: 43300 mM in grey matter, 35880 mM in white matter and 55009 mM in CSF. This value for CSF is based on the pure water density at 55556 mM (obtained by dividing 1 L or 1000 g of water by 18, the molecular weight of H^2O), but takes into account the higher physiological temperature (37°C) and the salt content (physiological solution containing approximately 9 g of NaCl per litre). This gives a density of 1000.001 g/ L (or 1000.001 Kg/ m^3), see http://www.csgnetwork.com/h2odenscalc.html).[S2] After subtracting the 9 g of salt we get 991.001 g of H^20 per litre which, divided by the molar mass of water (18.01528 mM), gives 55009 mM. The water signal was therefore corrected in each VOI using the following equation:

$$Water_{corr} = (fGM * wconcGM) + (fWM * wconcWM) + (fCSF * wconcCSF) \quad (1)$$

Where fGM is the fraction of grey matter in the voxel (as estimated by the Segment routine in SPM8) and wconcGM is the water concentration in grey matter, fWM and wconcWM are the respective values in white matter, and fCSF and wconcCSF are the respective values in CSF.

LCModel also uses an attenuation factor for the water signal related to the echo time (TE) used in the sequence and the tissue water T2.The default value in this attenuation factor accounts for water signal in white matter. This default value was substituted using a correction based on previously published T1 times (1122 ms in the hippocampus, cingulate and parietal cortices [S3], 986 ms in the thalamus, 758 ms in the white matter [S1] and 4300 ms in the CSF [S4-S5]) as well as T2 times (79 ms in the grey matter of the hippocampus, cingulate cortex and parietal cortex,67 ms in the thalamus, 81 ms in white matter [S3] and 2500 ms in CSF [S4-S5]).

Applying the parameter values used in this study (TE = 30 ms and TR = 6000 ms), and using the following standard equation relating MR signal to tissue relaxation times:

$$\text{Signal} \ \alpha \ (1 - \exp(-TR / T1)) * \exp(-TE / T2) \qquad (2)$$

this attenuation factor becomes 0.68078 in the hippocampus, cingulate and parietal cortices, 0.63760 in the thalamus, 0.69023 in the white matter and 0.74328 in the CSF.

Multiplying the NMR visible water concentrations in grey matter, white matter and CSF by these attenuation factors respectively, gives revised water content values of 29210 in the hippocampus, cingulate and parietal cortices, 27358 in the thalamus, 24528 in the white matter and 40887 in the CSF. These were substituted in place of LCModel's white matter default of 25516 for white matter voxels. Estimated metabolite concentrations thus corrected are reported in 'institutional units' (iu).

(2) Differences between cognitively impaired and cognitively preserved patients

28 % of patients (n = 5) were classified as cognitively impaired (CI), and 72 % (13) as cognitively preserved (CP) (**Table 1**).

CI patients showed 22 % lower [Glu] in the cingulate region compared to controls ($p < 0.01$), and 15 % lower [Glu] in the parietal region (which showed a trend, $p = 0.059$). CP patients showed 16 % lower [Glu] than controls in the cingulate ($p<0.01$) and 15 % lower [Glu] in the parietal region ($p < 0.01$). Similarly, a lower [Glx] in both cingulate and parietal GM regions (all $p < 0.01$) was found in both CI and CP patients than controls (**Table 2**).

(3) Additional analysis on [tNA] and its relationship with [Glu] and cognitive performance

Methods: In patients, the relationship between [tNA] and [Glu] levels was tested for each region using the Spearman's rho correlation coefficient. [tNA] was not normally distributed in the thalamus, and so non-parametric correlations were used for these analyses. The same findings are observed when using parametric (Pearson's) correlations. In order to assess whether [tNA] had a similar predictive value to [Glu], in cases in which a significant association between [Glu] levels and cognitive test performance was found, the regression analyses were repeated including [tNA] as the explanatory variable instead of [Glu].

Results: In patients, [Glu] concentration was significantly correlated with [tNA] in the hippocampus ($r_s = 0.80$, $p < 0.01$), cingulate ($r_s = 0.69$, $p < 0.01$), parietal ($r_s = 0.62$, $p < 0.01$), and thalamic regions ($r_s = 0.64$, $p < 0.01$).

In patients, the [tNA] concentration was not associated with cognitive performance in any of the VOIs where a significant association between [Glu] levels and cognitive performance was detected.

(4) Results on the differences in the concentrations of the main metabolites (except Glu and Glx).

Patients had significantly lower [tNA] than controls in the right thalamic, cingulate and parietal VOIs and lower [tCr] levels in the cingulate and parietal cortices (**Table 3**).

References

S1. Ernst T, Kreis R, Ross BD. Absolute quantification of water and metabolites in the human brain. I. Compartments and water. J Magn Reson B 1993; **102**: 1-8.

S2. McCutcheon SC, Martin JL, Barnwell TO. ”Water Quality”, in Maidment DR (Ed): “Handbook of Hydrology” McGraw-Hill, New York, NY 1993.

S3. Lu H, Nagae-Poetscher LM, Golay X, et al. Routine clinical brain MRI sequences for use at 3.0 Tesla. J Magn Reson Imaging 2005; **22**: 13-22.

S4. Rooney WD, Johnson G, Li X, et al. Magnetic field and tissue dependencies of human brain longitudinal 1H20 relaxation in vivo. Magn Reson Med 2007; **57**: 308-318.

S5. Schmitt P, Griswold MA, Jakob PM, et al. Inversion recovery TrueFISP: Quantification of T1, T2 and spin density. Magn Reson Med 2004; **51**: 661-7.

Table 1. Mean (and standard deviation) demographics and cognitive performance for controls, cognitively preserved and cognitively impaired patients.

**Demographic and clinical characteristics**	**Controls**	**Cognitively preserved**	**Cognitively impaired**
**N**	17	13	5
Age (SD, median, range)	39.7 (11.1, 40.0, 29-60)	43.6 (9.6, 46.0, 31 – 57)	43.2 (4.7, 43.0, 39 – 51)
Gender (M:F)	11:6		
EDSS (median [range])	-	2.5 (1.0 – 6.5)	3.5 (2.5 – 6.5)
Premorbid IQ	108.6 (11.7)	105.5 (12.7)	104.4 (16.7)
Current IQ	112.6 (16.8)	112.5 (10.7)	104.8 (17.5)
Anxiety	5.3 (2.8)	6.2 (3.9)	3.6 (3.6)
Depression	2.7 (3.5)	3.1 (2.2)	6.0 (4.6)
**Paired Associate Learning**			
Trials at 1st attempt (z-score)	0.2 (1.3)	-0.4 (0.6)	-1.7 (1.1)
Total trials (z-score)	0.2 (1.0)	-0.4 (0.6)	-1.8 (1.1)
**List learning**			
Learning (total words)	57.7 (9.0)	49.5 (9.4)	41.2 (6.1)
Recall after distraction (words)	12.2 (2.2)	9.4 (3.4)	8.6 (2.5)
30 min delayed recall (words)	11.8 (2.4)	10.4 (3.3)	7.8 (2.3)
**Digit span**	18.4 (3.1)	17.3 (3.5)	14.6 (1.3)
**Stroop (time to complete)**	101.7 (12.3)	141.5 (51.2)	170.4 (41.0)

**SDMT** (z-score)	0.9 (0.8)	-0.42 (1.0)	-2.1 (1.1)

Legend: EDSS: expanded disability status scale. SDMT: symbol-digit modalities test.

Table 2. Mean glutamate ([Glu]) and glutamate and glutamine ([Glx]) concentration estimates in institutional units in patients and controls.

	**Controls**	**Cognitively preserved**	**Cognitively impaired**
**Hippocampus**			
Glu	4.57 (1.2)	4.08 (1.1)	3.16 (0.3)
Glx	5.18 (1.5)	4.74 (1.6)	3.86 (0.04)
**Thalamus**			
Glu	4.99 (1.5)	4.98 (2.5)	4.36 (0.3)
Glx	5.84 (2.4)	6.45 (4.5)	5.08 (1.5)
**Cingulate cortex**			
Glu	7.59 (0.6)	6.34 (1.1)^	5.95 (1.9) ^
Glx	8.31 (0.9)	7.01 (1.2)^	6.77 (2.3)^
**Parietal cortex**			
Glu	7.43 (0.7)	6.38 (0.9)^	6.10 (1.7)°
Glx	8.49 (0.8)	7.28 (1.0)^	7.02 (1.8)

^ $P < 0.01$ for CP or CI vs. controls comparisons; °$p = 0.059$.

Table 3. Mean (standard deviation) corrected metabolite concentrations estimates (in institutional units) in patients and controls.

	**Controls**	**Patients**
**Hippocampus**		
[tNA]	4.26 (1.4)	4.18 (0.7)
[*m*Ins]	4.52 (1.0)	5.26 (1.1)
[Cho]	1.23 (0.3)	1.20 (0.3)
[tCr]	4.28 (1.3)	4.37 (0.9)
**Right thalamus**		
[tNA]	7.60 (1.0)	6.40 (1.5)**
[*m*Ins]	4.08 (0.9)	4.30 (2.2)
[Cho]	1.33 (0.2)	1.31 (0.3)
[tCr]	5.66 (0.9)	5.22 (1.3)
**Parietal cortex**		
[tNA]	8.18 (0.5)	7.39 (0.8)**
[*m*Ins]	5.28 (0.5)	5.04 (0.7)
[Cho]	0.94 (0.1)	0.83 (0.2)*
[tCr]	6.39 (0.4)	5.90 (0.5)**
**Cingulate cortex**		
[tNA]	8.33 (0.5)	7.34 (1.0)**
[*m*Ins]	5.45 (0.6)	4.99 (0.9)
[Cho]	1.17 (0.2)	1.03 (0.2)*

[tCr]	6.28 (0.4)	5.59 (0.8)**

* $p<0.05$; ** $p<0.01$
